# Innovative strategies targeting oral microbial dysbiosis: unraveling mechanisms and advancing therapies for periodontitis

**DOI:** 10.3389/fcimb.2025.1556688

**Published:** 2025-04-30

**Authors:** Yang Li, Xinyu He, Guocheng Luo, Juanjuan Zhao, Guohui Bai, Delin Xu

**Affiliations:** ^1^ Department of Medical Instrumental Analysis, Zunyi Medical University, Zunyi, China; ^2^ School of Stomatology, Zunyi Medical University, Zunyi, China; ^3^ Department of Immunology, Zunyi Medical University, Zunyi, China

**Keywords:** oral microbiome, microbial dysbiosis, periodontitis, mechanistic insights, therapeutic strategies inflammatory response

## Abstract

Periodontitis, a prevalent inflammatory oral disease, is intricately linked to disruptions in the oral microbiome, a state known as microbial dysbiosis. This review explores the pivotal roles of key pathogens, including *Porphyromonas gingivalis* and *Tannerella forsythia*, in driving periodontitis and examines the underlying molecular mechanisms that disrupt microbial homeostasis. We discuss how interactions among bacterial species affect the oral ecosystem’s balance and how microbial metabolites influence the host immune responses, contributing to disease progression. Leveraging these insights, we propose cutting-edge therapeutic approaches aimed at restoring microbial equilibrium. These include personalized pharmacological interventions tailored to individual microbiome profiles and innovative microbiome-targeted strategies such as probiotic formulations and bacteriophage therapy. By precisely modulating microbial communities, these strategies hold promise for enhancing treatment efficacy, preventing disease recurrence, and mitigating issues like antimicrobial resistance. Overall, this review paves the way for novel prevention and management techniques in periodontitis, offering significant improvements in oral health outcomes for patients.

## Graphical Abstract

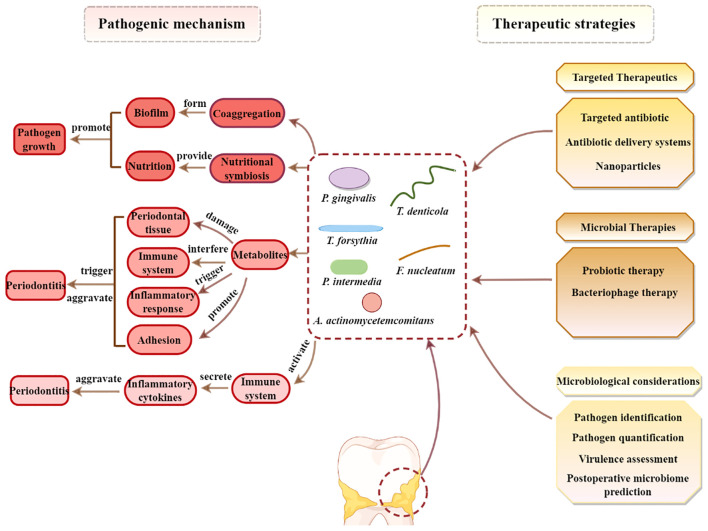

## 1 Introduction

The oral cavity constitutes a highly complex microbial ecosystem, hosting a diverse array of microorganisms. These microbes play a pivotal role in maintaining oral health and in the transition to disease states. The oral microbiome is the second most diverse after the gut, and this complex microbial community is crucial in balancing health and disease, providing essential metabolic functions and immune support for the host (Caselli et al., 2020). In a healthy state, a balanced relationship exists among these microbes, with mutual regulation maintaining oral homeostasis (Hussein et al., 2022; Santonocito et al., 2022). However, when this balance is disrupted, certain pathogenic microbes begin to overgrow, leading to a loss of biodiversity, which is a key factor in the development of oral diseases such as periodontal disease (Mensi et al., 2023).

Periodontitis is an extremely common chronic oral disorder that significantly impacts patients’ oral health. It is caused by a complex polymicrobial community within dental plaque and an imbalance in the oral microbial ecology, resulting from uncontrolled gingivitis (Hajishengallis et al., 2012). Affecting approximately 20-50% of the global population, it is characterized by the loss of periodontal tissue support, alveolar bone resorption, and gingival inflammation (Nazir et al., 2020). The formation of pathogenic biofilm around the teeth is a key factor in the occurrence of periodontitis. In the early stages of the disease, multiple bacteria synergistically act on the tooth surfaces, forming a complex biofilm structure (Tomasi et al., 2000). The bacteria embed and proliferate within the biofilm matrix, leading to plaque formation, which interacts with the host immune system to trigger an inflammatory response (Johnston et al., 2021). If left untreated, the persistent biofilm can result in tooth loss as the gingiva and alveolar bone recede from the tooth surface, leading to the formation of periodontal pockets as a consequence of periodontal ligament destruction (Sanz et al., 2017). The periodontal pocket provides an ideal hiding place for bacteria, making them more difficult to eliminate, thereby promoting the persistence and exacerbation of the inflammatory response.

Current conventional treatments for periodontitis primarily rely on mechanical debridement and antimicrobial agents (Sanz et al., 2020; Herrera et al., 2022). However, conventional treatments have limited efficacy and unclear long-term effects (Khattri et al., 2020). Thus, active treatment should be followed by supportive care to maintain periodontal health (Kwon et al., 2021). Accordingly, advancing our knowledge of the pathogenic mechanisms involved in periodontitis and developing new effective treatment strategies is critically necessary. This review summarizes the mechanisms of how microbial dysbiosis leads to periodontitis and the treatment approaches based on restoring microbial balance. The novelty of this review is manifested not only through an in-depth exploration of how main periodontal pathobionts contribute to the process of periodontitis but, more significantly, in the proposition of innovative therapeutic strategies aimed at restoring microbial equilibrium. By integrating targeted host microbiome interventions with conventional treatment modalities, this review aspires to offer a more comprehensive and enduring approach to the management of periodontitis. Furthermore, this review aims to enhance our understanding of the etiology of periodontitis, laying a solid foundation for preventive health management and the development of innovative treatment methodologies with wide-ranging clinical implications.

## 2 The microbial code of periodontitis

Under normal conditions, the oral cavity hosts a balanced symbiotic relationship among various microbial populations (Bacali et al., 2022). However, when the proportion of certain pathobionts (such as anaerobic Gram-negative bacteria) increases significantly, it leads to an imbalance in the oral microbial ecology (Li et al., 2022; Zhu et al., 2023) These pathobionts possess strong virulence and can trigger inflammatory responses. The major pathobionts identified include *Porphyromonas gingivalis (P. gingivalis*), *Tannerella forsythia (T. forsythia*), *Aggregatibacter actinomycetemcomitan (A. actinomycetemcomitan*), *Treponema denticola (T. denticola*), *Prevotella intermedia (P. intermedia*) and *Fusobacterium nucleatum (F. nucleatum*). These organisms are associated with the development of periodontitis under dysbiotic conditions.

### 2.1 P. gingivalis

**Figure 1:**
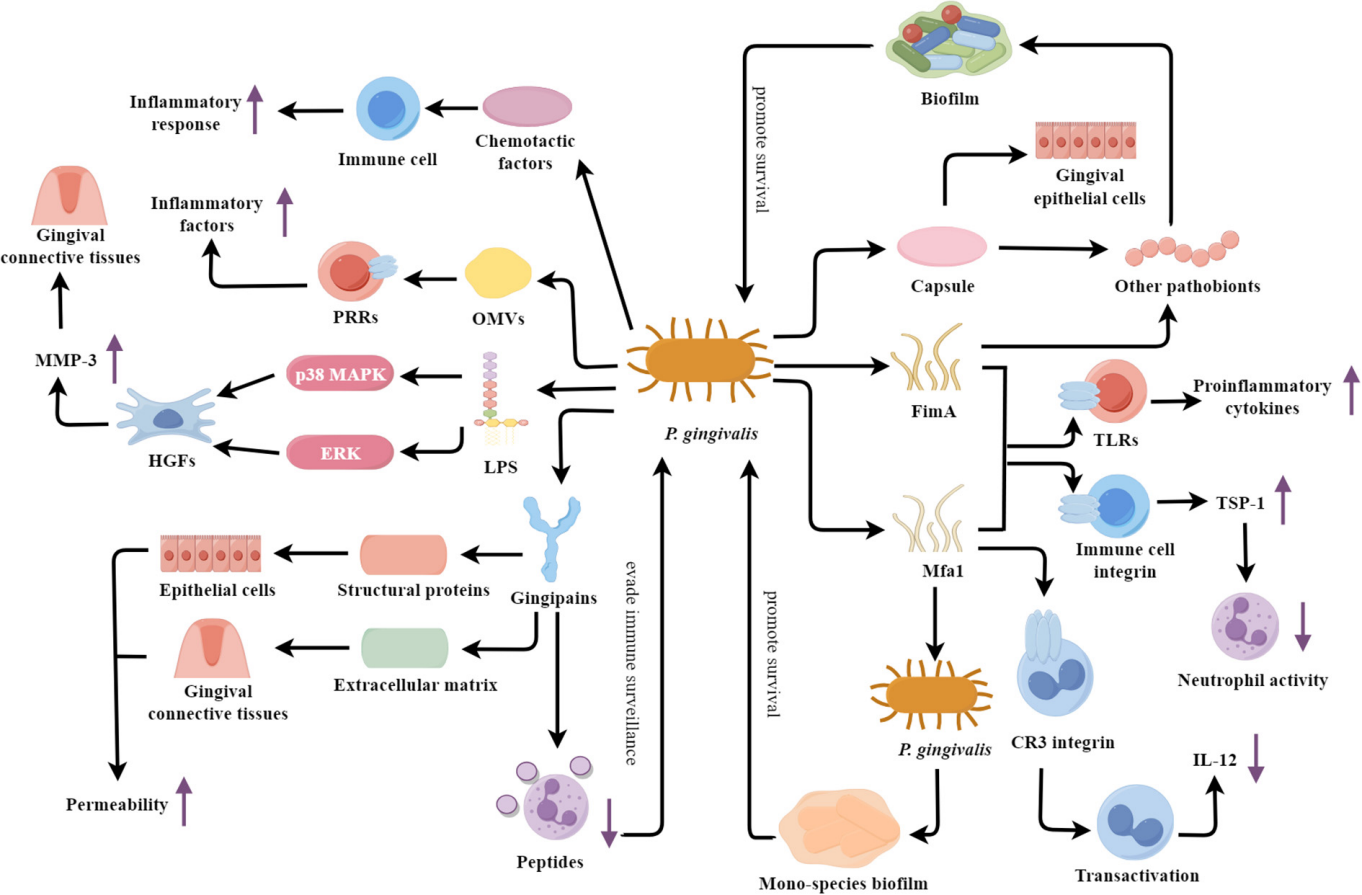
*P. gingivalis*: a veritable arsenal of virulence factors fueling periodontal destruction. TLRs, Toll-like receptors; LPS, Lipolyaccharide; OMVs, Outer membrane vesicles; HGFs, Human gingival fibroblasts; MMP-3, Matrix metalloproteinase-3.

*P. gingivalis* is an anaerobic, Gram-negative, short-rod bacterium and is recognized as the primary etiological agent of periodontitis (Torrungruang et al., 2015). *P. gingivalis* exerts its pathogenicity through various virulence factors (**Figure 1**), which play crucial roles in the initiation and progression of periodontal disease.

One of the key virulence factors is the bacterial fimbriae, including FimA and Mfa1. These fimbrial structures mediate the adhesion and invasion of *P. gingivalis* into host epithelial and immune cells (Hasegawa and Nagano, 2021). Recent studies have elucidated the structural basis of FimA and Mfa1 interaction with host cell receptors, revealing a sophisticated mechanism of host manipulation and immune evasion. FimA and Mfa1 fimbriae can induce immune responses via interaction with Toll-like receptors (TLRs), resulting in proinflammatory cytokine production (Hajishengallis et al., 2006; Hasegawa and Nagano, 2021). They can also engage with host immune cell integrins, initiating a signaling pathway that culminates in TSP-1 secretion (Angabo et al., 2024). TSP-1 then inhibits neutrophil activity by decreasing ROS levels, ultimately improving bacterial survival (Angabo et al., 2024). In addition, FimA fimbriae can mediate the binding of *P. gingivalis* to other pathobionts, such as *Actinomyces* and *Prevotella* spp., then promoting biofilm formation (Goulbourne and Ellen, 1991; Yamada et al., 2005). Meanwhile, Mfa1 fimbriae participate in the self-aggregation of *P. gingivalis* and regulate the formation of mono-species biofilms (Ikai et al., 2015). This self-aggregation is now understood to be a critical step in biofilm maturation, significantly enhancing resistance to antimicrobial agents. Mfa1 can bind to the complement receptor 3 (CR3) integrin on the surface of macrophages, inducing their transactivation and suppressing the production of interleukin-12 (IL-12) (Hajishengallis, 2009; Hajishengallis et al., 2013). FimA can promote the entry of *P. gingivalis* into macrophages and inhibit the production of the key immune factor IL-12p70 through its interaction with host TLR2 and CR3 receptors (Wang et al., 2007).

Another important virulence factor is the secreted gingipains, which can disrupt the host cell-cell junctions and extracellular matrix. The gingipains of *P. gingivalis* mainly include arginine-specific gingipains (RgpA, RgpB) and lysine-specific gingipains (Kgp) (Kadowaki, 2021). These enzymes are capable of hydrolyzing the structural proteins of the host, compromising the tight junctions between epithelial cells and increasing the permeability of the epithelial barrier, thus providing an easier route for *P. gingivalis* to invade the host tissues (Genco et al., 1999; Bostanci and Belibasakis, 2012). Furthermore, the gingipains can also degrade components of the extracellular matrix, such as cell adhesion proteins and collagen, disrupting the integrity of the gingival connective tissues, amplifying the inflammatory response, and promoting the deeper invasion of *P. gingivalis* into the host (Genco et al., 1999; Bostanci and Belibasakis, 2012). Additionally, the gingipains can hydrolyze the antimicrobial peptides of neutrophils, suppressing the microbicidal functions of these immune cells and enabling *P. gingivalis* to evade host immune surveillance (Bryzek et al., 2019). These gingipains can also cleave and inactivate key complement components C3 and C5, thereby blocking the complement cascade reaction and achieving immune evasion (Popadiak et al., 2007). In addition, RgpA specifically binds to the complement inhibitory protein C4b-binding protein through its unique hemagglutinin-adhesin domain, further preventing the deposition of the membrane attack complex on the surface of *P. gingivalis*, protecting the bacteria from direct complement attacks (Potempa et al., 2008).

The lipopolysaccharide (LPS) of *P. gingivalis* is also a key contributor to the pathogenesis of periodontitis. The penta-acylated *P. gingivalis* LPS (LPS1690) significantly upregulates MMP-3 expression in HGFs through the activation of p38 mitogen-activated protein kinase (MAPK) and extracellular signal-regulated kinases (ERK) signaling pathways (Herath et al., 2013). The dysregulated expression of MMP-3 may disrupt the homeostatic balance of the gingival connective tissues and facilitate the chronic inflammatory response (Razavi et al., 2023). Additionally, the outer membrane vesicles (OMVs) secreted by *P. gingivalis* can carry multiple virulence factors and bind to the pattern recognition receptors (PRRs) of host cells, activating immune cells and inducing the release of large amounts of inflammatory factors (Uemura et al., 2022). *P. gingivalis* also possesses another crucial virulence factor—capsule. The capsule structure not only facilitates the binding of *P. gingivalis* with other pathobionts (such as *F. nucleatum*) to form biofilms, but also enhances the adhesion of *P. gingivalis* to gingival epithelial cells. These properties contribute to the persistent infection of *P. gingivalis* (Dierickx et al., 2003; Rosen and Sela, 2006). Chemotactic factors released by *P. gingivalis* significantly influence the progression of periodontitis. For instance, research by Rudin et al (Dahlstrand Rudin et al., 2020). has identified various water-soluble chemotactic factors, including short-chain fatty acids, which effectively recruit and activate neutrophils into the periodontal pocket via the FFAR2 receptor-mediated signaling pathway, thereby enhancing local inflammatory responses. The peptidylarginine deiminase (PPAD) secreted by *P. gingivalis* also plays a significant role in the progression of periodontitis. PPAD not only stimulates neutrophils to secrete abnormal levels of IL-6 and TNF-α but also affects the generation of ROS and phagocytic activity in neutrophils, thereby enhancing the survival of *P. gingivalis* (Prucsi et al., 2023).

### 2.2 T. forsythia

**Figure 2:**
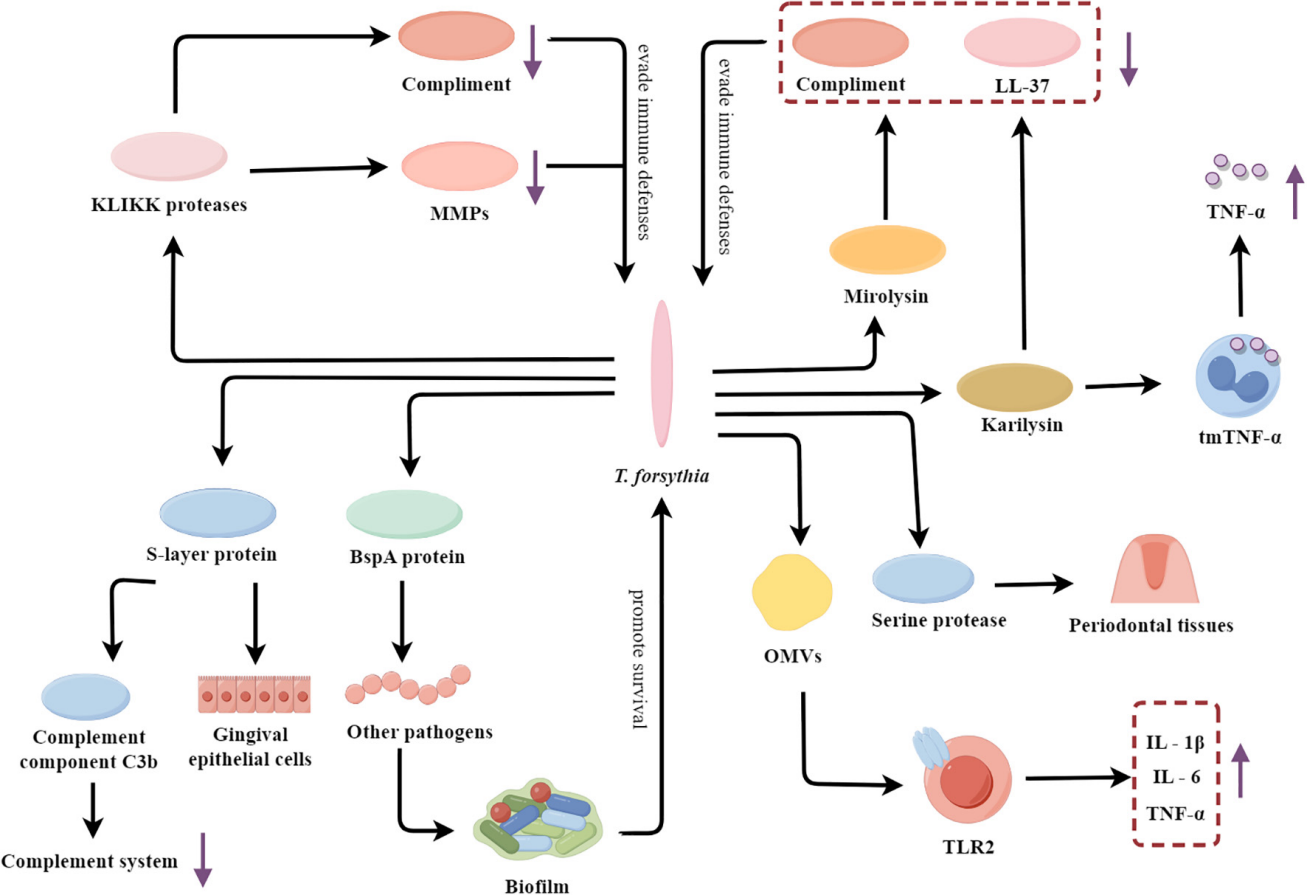
*T. forsythia’*s multifaceted arsenal: fueling the flames of periodontal inflammation. OMVs, Outer membrane vesicles; TLR2, Toll-like receptor 2; tmTNF-α, The membrane-bound form of TNF-α; MMPs, Matrix metalloproteinases.

*T. forsythia* is a Gram-negative, anaerobic, spindle-shaped bacterium that is considered a key pathobiont responsible for periodontitis in humans (Kirst et al., 2015; Hottmann et al., 2021). *T. forsythia* is highly prevalent in subgingival biofilms of patients with advanced periodontal disease and its abundance is strongly correlated with disease severity (Bloch et al., 2017). It employs a multifaceted arsenal of virulence factors to subvert host defenses and drive the pathogenesis of periodontitis (**Figure 2**).

*T. forsythia* contains a variety of virulence factors such as surface-layer (S-layer) protein, *Bacteroides* surface protein A (BspA), and KLIKK protease, which enhance its virulence and adaptability to the host environment. The S-layer protein can mediate the specific adhesion of the bacteria to the human gingival epithelial cells, enhancing the affinity of the bacteria to the human gingival epithelial cells (Sakakibara et al., 2007; Philips et al., 2020). Furthermore, the S-layer protein plays a crucial role in the immune evasion strategies of *T. forsythia*. It has been shown to inhibit the deposition of complement component C3b onto the surface of *T. forsythia* cells, thereby suppressing the complement activation pathway to resist complement-mediated bactericidal action (Shimotahira et al., 2013). A key virulence strategy of *T. forsythia* involves the evasion and interference of host immune defenses. The BspA protein promotes the formation of more pathogenic multispecies biofilms through mediating interspecies interactions (Sharma, 2010). The KLIKK proteases secreted by *T. forsythia* not only cleave and inactivate complement proteins to evade complement-mediated killing, but also specifically inhibit MMPs, which are critical components of the host’s antimicrobial arsenal (Koziel et al., 2010; Książek et al., 2023). Among the KLIKK proteases, mirolysin and karilysin are the primary enzymes that play significant pathological roles. Mirolysin and karilysin can cleave complement components and degrade the antimicrobial peptide LL-37 (Skottrup et al., 2019; Radisky, 2020). And karilysin has been demonstrated that it can cleave the membrane-bound form of tumor necrosis factor-α (TNF-α) on the surface of macrophages, leading to the release of the pro-inflammatory cytokine TNF-α and triggering an inflammatory response (Bryzek et al., 2014). This cleavage amplifies inflammation and contributes to tissue destruction and bone resorption, leading to chronic periodontitis. *T. forsythia* can also secrete another protease which is serine protease, an enzyme that directly cleaves gelatin and type I collagen, thus disrupting the structural integrity of periodontal tissues (Hockensmith et al., 2016). In addition, *T. forsythia* also actively induces a dysregulated inflammatory response by releasing OMVs containing many virulence factors. These OMVs activate TLR2 signaling in host cells and then induce host cells to produce excessive inflammatory mediators (IL‐1β, IL‐6, and TNF-α) by activating the TLR2 pathway (Lim et al., 2023). The LPS of *T. forsythia* is also capable of inducing the release of pro-inflammatory factors. For instance, its LPS significantly induces the release of the chemokine IP-10 in macrophages through the activation of the TLR4 and STAT1 signaling pathways (Chinthamani et al., 2022). This uncontrolled inflammatory response eventually leads to severe damage and degradation of periodontal tissue.

### 2.3 A. actinomycetemcomitans

**Figure 3:**
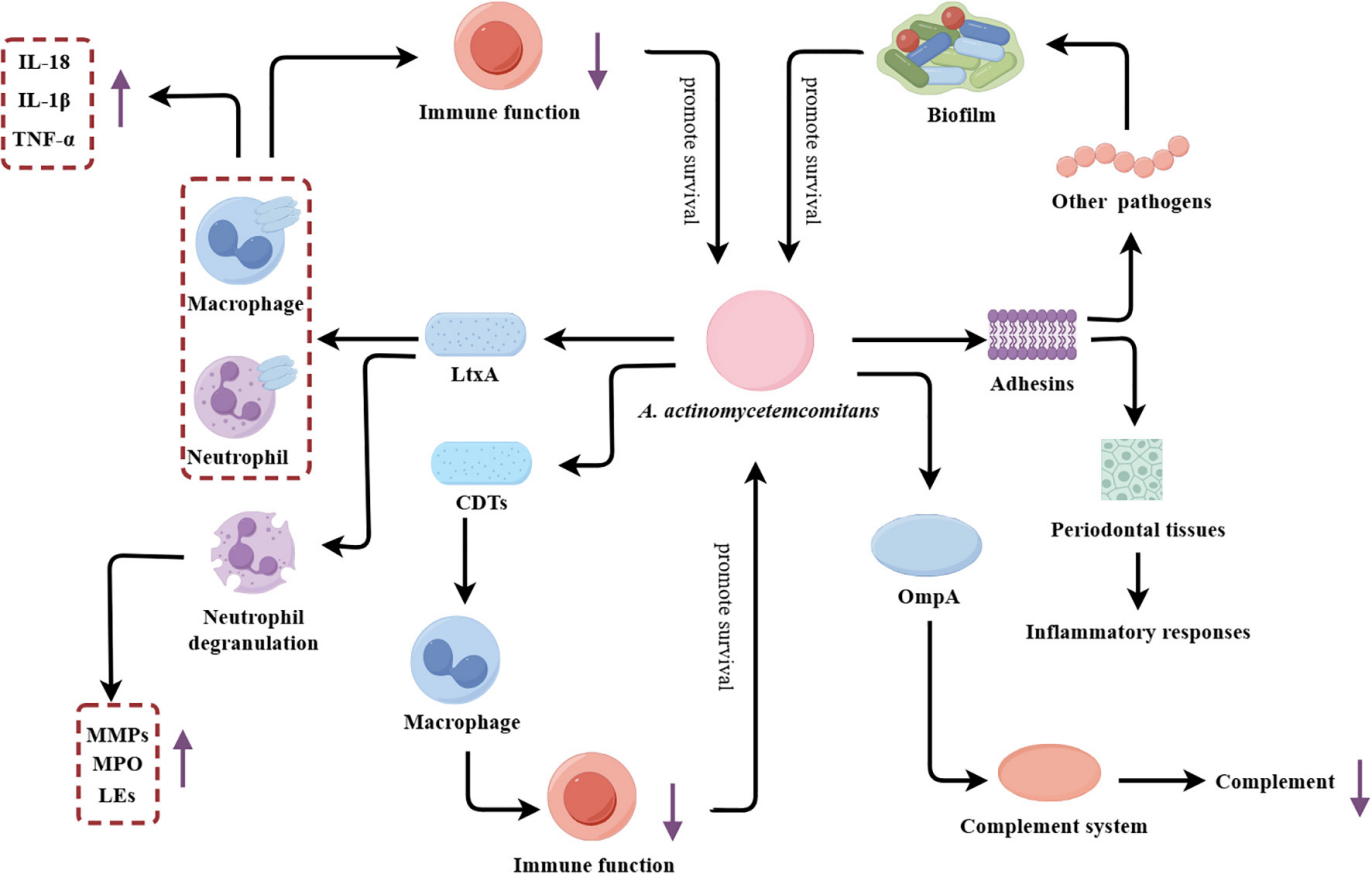
*A. actinomycetemcomitans*’s multifaceted virulence factors driving periodontal pathogenesis. LtxA, Leukotoxin A; CDTs, Cytotoxic distending toxins; MMPs, Matrix metalloproteinases; MPO, Myeloperoxidase; Les, Lysosomal enzymes; OmpA, Outer membrane protein A.

*A. actinomycetemcomitans* is a key etiological agent of periodontitis in adolescents, and its pathogenicity has been extensively studied (Fine et al., 2007). This Gram-negative, facultative anaerobic coccobacillus utilizes a multifaceted arsenal of virulence factors to subvert host immunity and exacerbate periodontal tissue destruction (**Figure 3**).

The principal virulence determinant of *A. actinomycetemcomitans* is repeats in toxin (RTX) toxin. The RTX toxin of *A. actinomycetemcomitans* is leukotoxin (LtxA), which plays a pivotal role in the pathogenesis of this organism. LtxA is a potent leukotoxin that selectively disrupts host immune cells, inducing both humoral and cellular immune responses, and ultimately amplifying tissue damage (Johansson, 2011; Johansson et al., 2017). Mechanistically, LtxA facilitates the degradation of type I collagen in periodontal tissues by inducing degranulation and dissolution of neutrophils, thereby promoting the release and activation of MMP-8 (Claesson et al., 2002). In addition to MMP-8, LtxA has been demonstrated to facilitate the release of other substances by inducing neutrophil degranulation, including lactoferrin and elastase, which contribute to the destruction of periodontal tissue (Johansson et al., 2000). LtxA also impairs host immune function by recognizing and binding to cells expressing human lymphocyte function-associated antigen 1 (CD11a/CD18), inducing lysosomal damage and causing cytoplasmic acidification (Balashova et al., 2016). Moreover, LtxA can activate inflammatory signaling pathways in immune cells, leading to the production of inflammatory cytokines. For instance, previous research has demonstrated that LtxA can induce human macrophages to release a significant amount of the pro-inflammatory cytokine IL-1β through the activation of caspase-1 (Kelk et al., 2005).

Beyond the RTX toxin, *A. actinomycetemcomitans* possesses additional virulence factors that amplify its pathogenic effects. Similar to other pathobionts, *A. actinomycetemcomitans* enhances its adaptability by forming biofilms. The diverse array of adhesins such as EmaA and Aae expressed on the bacterial surface promote adherence to other microbes, further facilitating biofilm formation (Danforth et al., 2021). In addition to LtxA, *A. actinomycetemcomitans* can also secrete another toxin, namely cytolethal-distending toxins (CDTs). CDTs can impair macrophage function by disrupting phagosome maturation and phagolysosome formation, thereby inhibiting the clearance of *A. actinomycetemcomitans* (Shenker et al., 2022; Kim et al., 2023). Additionally, the secreted outer membrane protein OmpA, which activates the complement system and consumes complement components, allowing the bacterium to evade host humoral immunity and enhance antibiotic tolerance (Lindholm et al., 2019; Lindholm et al., 2020).

### 2.4 T. denticola

**Figure 4:**
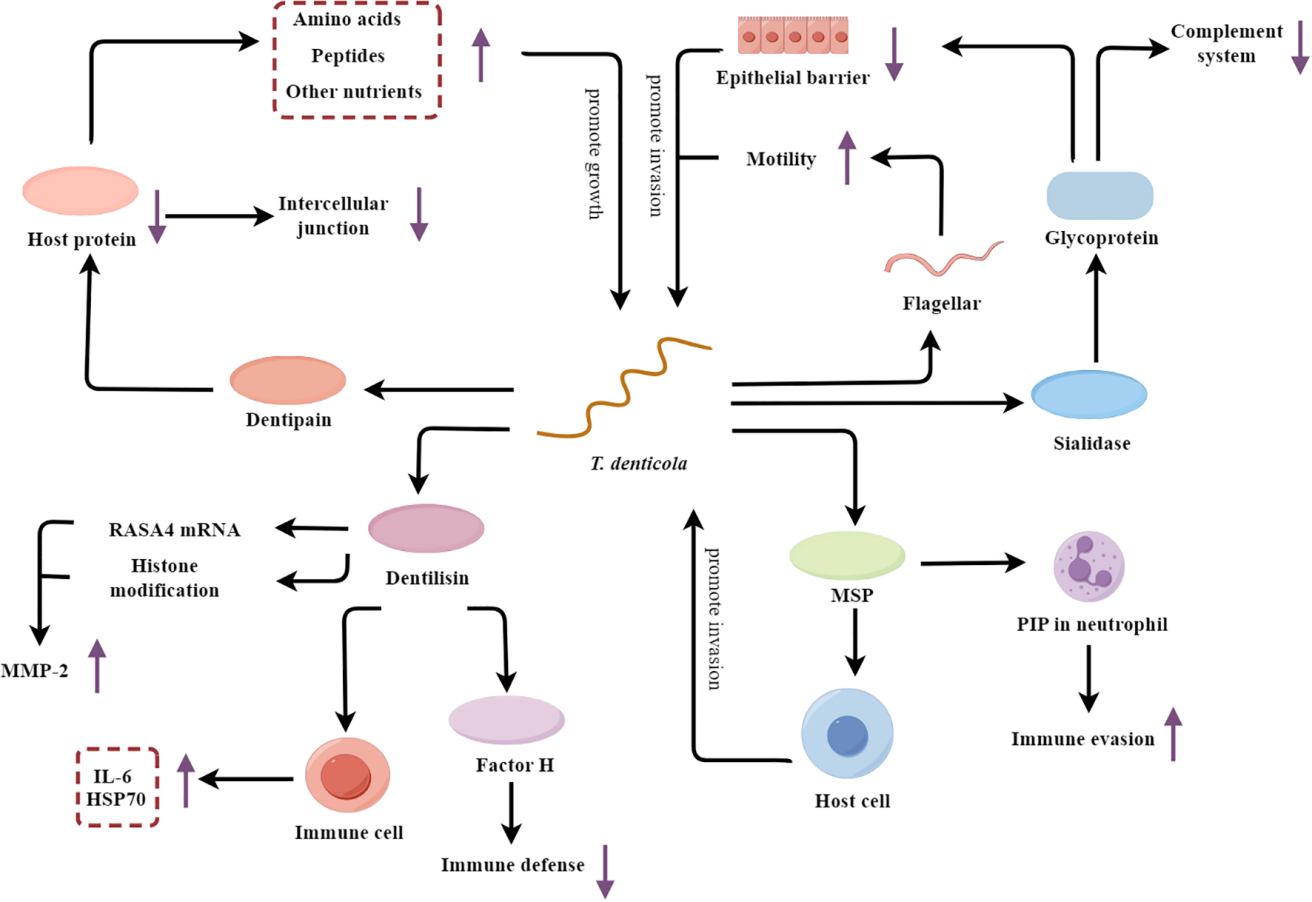
*T. denticola*’s multifaceted mechanisms fueling periodontal pathogenesis. MSP, major surface protein; PIP, Phosphoinositide processing; MMP-2, Metalloproteinase-2; HSP70, Heat shock protein 70.

*T. denticola* is a Gram-negative, anaerobic spirochete that is widely recognized as a key etiological agent of periodontitis (Ng et al., 2019; Goetting-Minesky et al., 2021). *T. denticola* employs a sophisticated arsenal of virulence factors to subvert host defenses and promote the development and progression of destructive periodontal disease (**Figure 4**).

*T. denticola* secretes sialidase, which hydrolyzes sialic acid from host glycoproteins at the tight junctions between epithelial cells, thereby compromising the integrity of the epithelial barrier and facilitating the invasion of host tissues (Frey et al., 2019). The hydrolysis of sialic acid not only disrupts host tissues, but also releases hydrolyzed sialic acid as a nutritional source for *T. denticola*’s growth (Kurniyati et al., 2013). Furthermore, *T. denticola* alters the glycosylation patterns on the host cell surfaces by hydrolyzing sialic acid, thereby interfering with the function of several key complement factors, such as C1q and Factor H (Kurniyati et al., 2013). This interference weakens the activity of the complement system, enhancing the bacterium’s survival rate. Except for sialidase, the flagellar motility of *T. denticola* enhances its ability to penetrate the epithelium and access the underlying periodontal tissues (Kurniyati et al., 2017). Glycosylation at specific sites on flagellin (such as at the S116 position) may interfere with the interaction between flagellin and TLR5 in the host immune system, facilitating bacterial evasion of the host immune response (Kurniyati et al., 2017).

In addition to breaching the epithelial barrier, *T. denticola* also actively adheres to and invades the host cells. The dentipain protease secreted by the bacterium promotes its adhesion and facilitates the acquisition of essential nutrients (Miyai-Murai et al., 2023). Dentipain is a cysteine protease that can degrade a variety of host proteins, including those involved in cell-cell junctions and the extracellular matrix (Ishihara et al., 2010; Miyai-Murai et al., 2023). By degrading these host proteins, dentipain allows *T. denticola* to more easily adhere to and invade the host epithelial and connective tissues. Additionally, the breakdown of host proteins provides *T. denticola* with a source of amino acids, peptides, and other nutrients that the bacterium can utilize for its growth and proliferation within the periodontal environment. Besides the dentipain, the major surface protein (MSP) of *T. denticola* also synergistically enhances the bacterium’s adherence and invasive capabilities (Godovikova et al., 2019; Jones et al., 2019). Interestingly, MSP also contributes to immune evasion by blocking phosphoinositide processing (PIP) in neutrophils, thereby impairing their phagocytic and microbicidal functions (Jones et al., 2019). Furthermore, *T. denticola* secretes the protease dentilisin, which upregulates the expression of MMP-2 through mechanisms involving RAS p21 protein activator 4 (RASA4) mRNA expression or histone modification (Ateia et al., 2018; Malone et al., 2021). This leads to the degradation of the extracellular matrix and the destruction of periodontal tissues. The dentilisin also stimulates host immune cells, causing these cells to produce more inflammatory mediators IL-6 and heat shock protein 70 (HSP70), thus exacerbating the inflammatory response (Kokubu et al., 2018). Dentilisin also degrades complement proteins such as factor H, thereby diminishing the effectiveness of innate immune defenses (Miller et al., 2016).

### 2.5 P. intermedia

**Figure 5:**
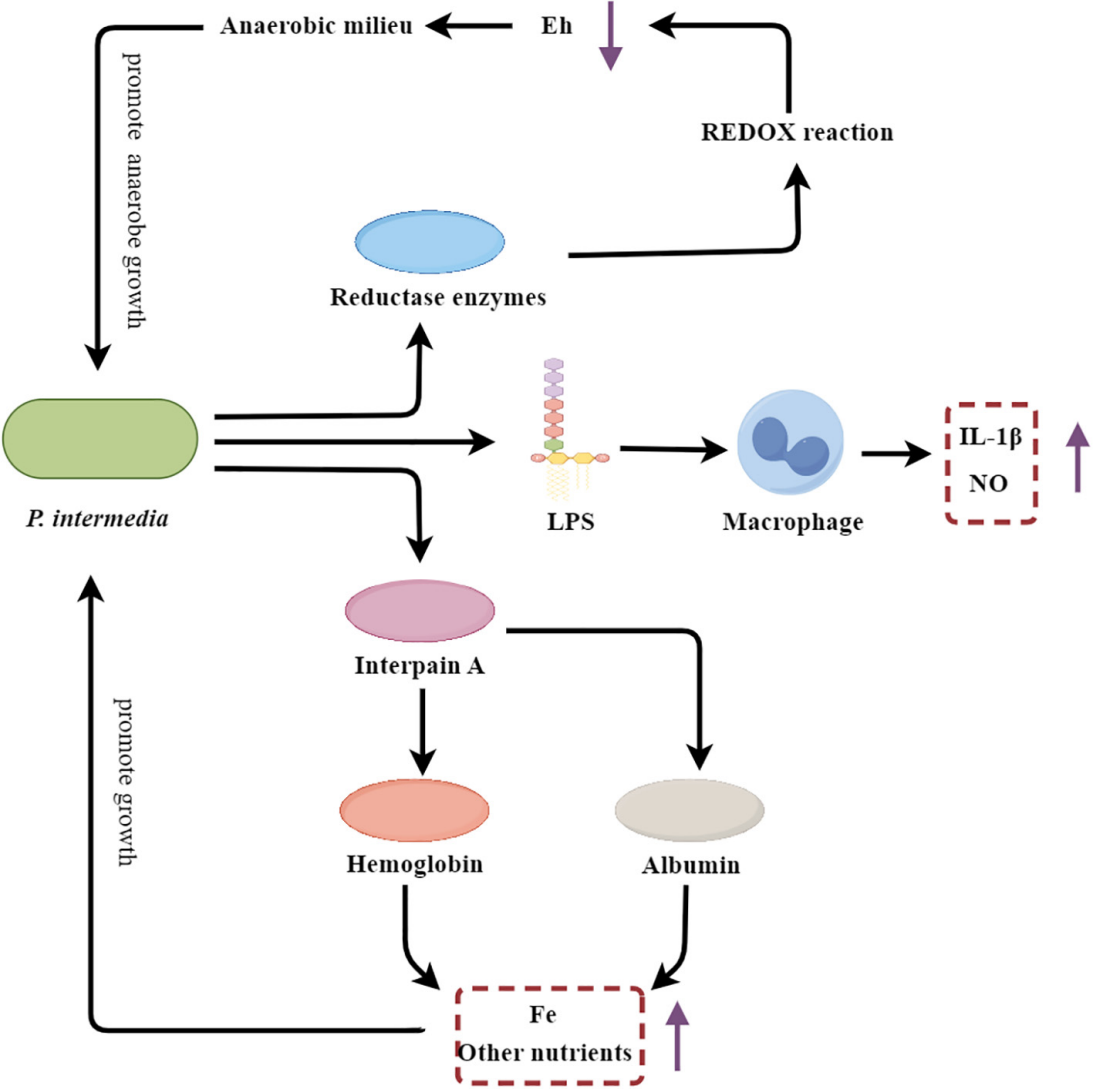
*P. intermedia*’s multifarious strategies fueling periodontal inflammation and destruction. Eh, REDOX potential; LPS, Lipopolysaccharide.

*P. intermedia* is an anaerobic, Gram-negative short-rod bacterium that is strongly associated with the development and progression of chronic periodontitis (Petrović et al., 2018). It contributes to the initiation and progression of periodontitis through various mechanisms (**Figure 5**).

*P. intermedia* contributes to the initiation of the inflammatory cascade through the activation of host immune cells. The LPS component of the *P. intermedia* cell wall stimulates macrophages to produce pro-inflammatory mediators, such as NO and IL-1β, thereby amplifying the inflammatory response within the periodontal tissues (Choe et al., 2021). Additionally, *P. intermedia* secretes a cysteine protease known as interpain A, which degrades host proteins, including albumin and hemoglobin (Byrne et al., 2015). The resulting albumin fragments can serve as a nutrient source, particularly for iron, which is essential for the growth and proliferation of *P. intermedia* and other oral pathobionts (Byrne et al., 2015). Moreover, interpain A-mediated degradation of hemoglobin releases heme, a critical iron source that supports the growth of the anaerobic microbial community within the periodontal pocket (Byrne et al., 2015). Beyond its direct proinflammatory and nutrient-scavenging capabilities, *P. intermedia* also plays a crucial role in shaping the local periodontal environment to favor the establishment and persistence of anaerobic pathobionts. This bacterium secretes various reductase enzymes, including superoxide reductase, which lower the redox potential (Eh) within the periodontal pocket, creating a more anaerobic milieu that selectively promotes the growth of anaerobic species (Karched et al., 2022). Furthermore, *P. intermedia* has been shown to suppress the phagocytic activity of host neutrophils through the inhibition of phagosome maturation and the reduction of ROS production, thereby diminishing the production of oxygen radicals and further contributing to the development of an anaerobic environment (Doke et al., 2017).

### 2.6 F. nucleatum

**Figure 6:**
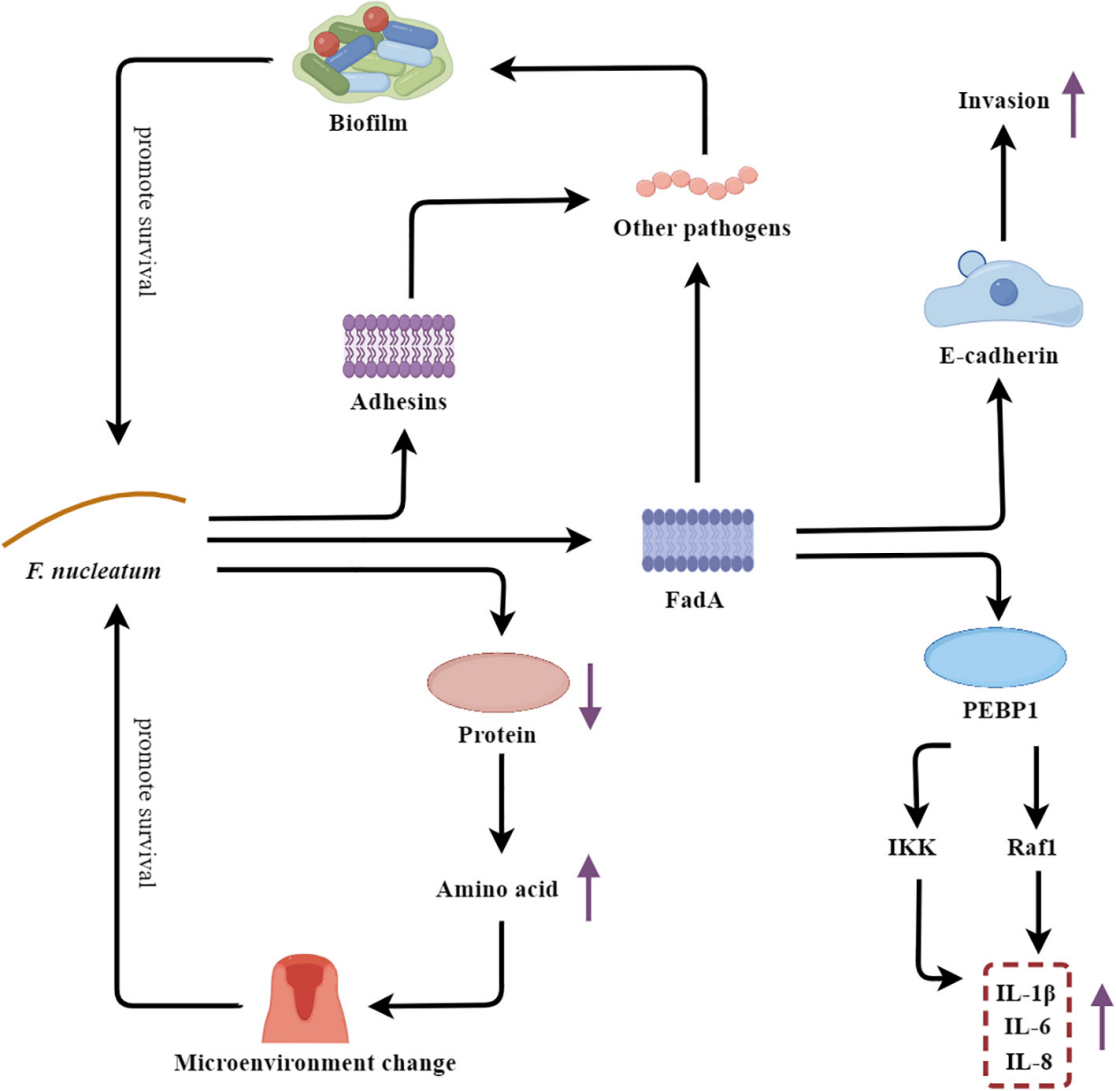
*F. nucleatum’s* multifaceted strategies fueling the vicious cycle of periodontal disease. PEBP1, Cytoplasmic protein phosphatidylethanolamine-binding protein 1.

*F. nucleatum* is an anaerobic, Gram-negative, fusiform-shaped bacterium that is widely recognized as a major etiological agent of periodontal disease (Song et al., 2023). *F. nucleatum* employs a multifaceted approach to promote the initiation and progression of destructive periodontitis (**Figure 6**).

As an adhesive bacterium, *F. nucleatum* plays a crucial role in maintaining the integrity of the polymicrobial biofilms associated with periodontal disease. One of the key virulence factors of *F. nucleatum* is the adhesin FadA, which facilitates the bacterium’s colonization and invasion of the host. FadA binds to E-cadherin on the surface of oral epithelial cells, enabling *F. nucleatum* to adhere to and penetrate the epithelial barrier (Meng et al., 2021). Moreover, FadA also promotes the adhesion of *F. nucleatum* to other pathobionts, contributing to the formation of a stable and cohesive biofilm structure within the dental plaque (Li et al., 2020; Meng et al., 2021). In addition to FadA, *F. nucleatum* also expresses other adhesins, such as RadD and Fap2, which further enhance the bacterium’s ability to interact with and integrate into the polymicrobial biofilm community (Coppenhagen-Glazer et al., 2015; Shokeen et al., 2020). The formation of this complex biofilm matrix confers increased resistance to host defenses and antimicrobial agents, allowing *F. nucleatum* to thrive in the adverse periodontal environment. Additionally, the FadA adhesin of *F. nucleatum* also functions as a potent virulence factor by triggering a robust host immune response. FadA binds to the cytoplasmic protein phosphatidylethanolamine-binding protein 1 (PEBP1), leading to the activation of the Raf1 and IKK signaling pathways, which in turn activate the ERK-JNK-p38 MAPK and NF-κB-p65 cascades. This signaling culminates in the production of a range of proinflammatory mediators (IL-1β, IL-6, and IL-8), thereby amplifying the inflammatory response within the periodontal tissues (Wang Y. et al., 2023). Furthermore, *F. nucleatum* is capable of hydrolyzing proteins and generating an abundance of amino acid metabolites, which can alter the local oral microenvironment, such as increasing the pH and redox potential. These changes in the periodontal milieu create conditions that favor the growth and proliferation of other pathogenic microorganisms, thereby contributing to the dysbiosis and progression of periodontal disease (Wang M. et al., 2023; Hara et al., 2024).

## 3 Dysbiosis and periodontitis

**Figure 7:**
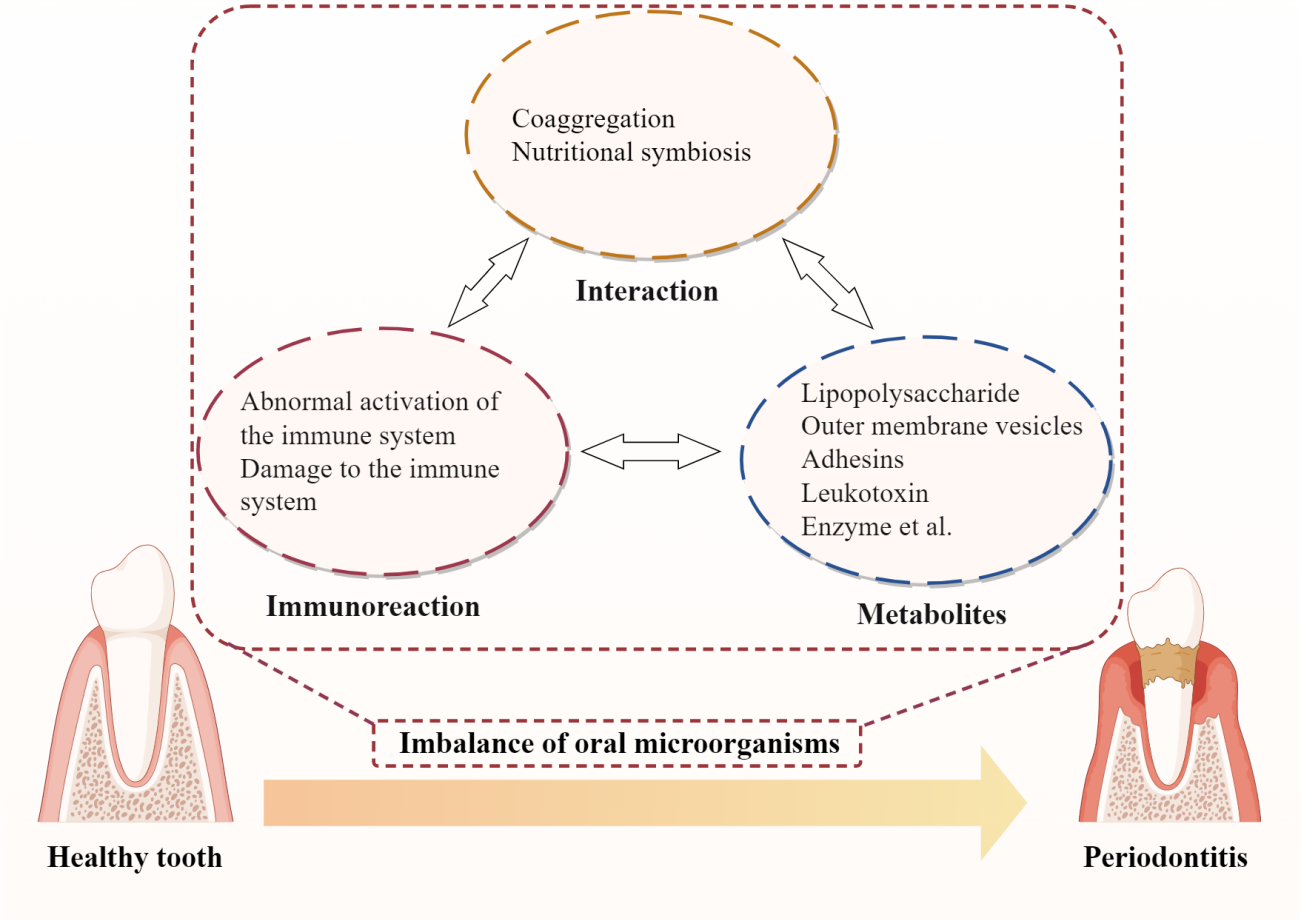
Vicious cycles of periodontal pathogenesis: microbial interaction, host immunity and bacterial virulence.

Dysbiosis, an imbalance in the microbial community, is a key factor in the development of periodontitis. Periodontopathic bacteria initiate and perpetuate periodontitis through various mechanisms (**Figure 7**). These mechanisms involve bacterial interactions, host immune responses, and the production of bacterial virulence factors, which interact and form a vicious cycle, ultimately triggering and aggravating periodontitis, leading to the progressive destruction of periodontal tissues (Santos et al., 2020; Xu et al., 2020; Teles et al., 2022).

### 3.1 Microbial interactions in Periodontitis

The oral cavity is a complex microbial ecosystem, harboring a diverse range of microorganisms. In a healthy state, these microbes coexist in a balanced equilibrium, maintaining oral health. However, when this balance is disrupted, an overgrowth of certain pathobionts can lead to the development of periodontitis (Łasica et al., 2024).

Microbial interactions play a significant role in the progression of periodontitis. One such interaction is coaggregation, where certain bacteria secrete signaling molecules that promote the aggregation and growth of other pathobionts, collectively forming a resilient dental plaque biofilm (Valm, 2019; Jakubovics et al., 2021; Qiao et al., 2023). For instance, *P. gingivalis* is capable of establishing biofilms in conjunction with pathobionts such as *Actinomyces* and *Prevotella* spp., facilitated by its fimbriae (Goulbourne and Ellen, 1991; Yamada et al., 2005). These biofilms provide a protective environment, shielding both *P. gingivalis* and other pathobionts from the host immune system and antibiotic treatments. A critical determinant in the successful colonization of oral pathobionts is their capacity to form stable biofilm structures in conjunction with other microbial communities present in the oral cavity. A successful establishment of *P. gingivalis* in the oral environment relies on its ability to interact with primary colonizers within the dental biofilm, such as *Streptococcus oralis* and *Streptococcus gordonii* (Lamont et al., 1993; Kuboniwa et al., 2009). Similarly, the ability of *T. forsythia* to successfully colonize the oral environment is predicated upon its synergistic interactions with other pathobionts within the red complex, such as *P. gingivalis* and *T. denticola* (Jiang et al., 2021). Additionally, quorum sensing mechanisms allow bacteria to communicate and coordinate their activities, enhancing biofilm robustness. Research conducted by Su et al (Su et al., 2023). has demonstrated that quorum-sensing signaling molecules promote the aggregation and information exchange among key microbial communities such as *Streptococcus* and the *Prevotella*-*Fusobacteria* complex, which can enhance the structural stability and resilience of biofilms. Moreover, QS-mediated metabolic cooperation and environmental adaptability enable pathogens to establish an ecological niche conducive to their survival and proliferation.

Another type of microbial interaction is nutritional symbiosis, where bacteria exchange metabolic products provide essential nutrients for bacteria’s growth. For example, amino acids constitute a significant source of energy for *F. nucleatum*’s growth. *F. nucleatum* is capable of engaging in nutritional complementation and metabolic product exchange with bacteria possessing proteolytic capabilities, such as *P. gingivalis*, *Streptococcus gordonii*, and *Veillonella parvula* (Sakanaka et al., 2022). This interaction facilitates the effective acquisition of the requisite amino acids. *P. gingivalis* degrades hemoglobin to produce heme, which is utilized by *Actinomyces* for growth (Zhang L. et al., 2020; Śmiga et al., 2024). *Veillonella* utilizes lactic acid produced by *Streptococcus* and *Actinomyces* for its metabolism, while *Streptococcus* and *Actinomyces* use acetic acid produced by *Veillonella* as a carbon source (Scheiman et al., 2019; Zmysłowska-Polakowska et al., 2023). This metabolic exchange not only supports bacterial proliferation but also creates a suitable environment conducive to pathogenicity. *F. nucleatum* is able to enhance the availability of ornithine in co-culture with *Streptococcus gordonii*, thereby facilitating putrescine production (Sakanaka et al., 2022). This metabolic augmentation not only creates a putrescine-rich microenvironment on the surface of the *F. nucleatum* biofilm but also alters the biofilm phenotype of *P. gingivalis*, accelerating its transition from a planktonic state to a mature biofilm (Sakanaka et al., 2022). Additionally, *P. intermedia* secretes a variety of reductases, including superoxide reductase, which reduce the Eh within the periodontal pocket, thereby creating a more anaerobic environment (Karched et al., 2022). Such an environment not only selectively promotes the growth of anaerobes such as *P. gingivalis*, but also creates favorable conditions for the development of pathogenicity.

### 3.2 Microbial metabolites and pathological changes in periodontal tissues

Microbial metabolites are closely associated with pathological changes in periodontal tissues. Pathogenic bacteria in the oral cavity produce various toxic substances, such as enzymes and endotoxins, through their metabolic activities. These substances can directly compromise the integrity of periodontal tissues and indirectly cause damage via the induction of the host’s immune response. **Table 1** summarizes the major pathobionts and the mechanisms of action of their virulence factors. The metabolic products of these microorganisms have potent biological activities that directly stimulate gingival tissue, initiating a local inflammatory response.

**Table 1:** Major pathobionts and the mechanisms of action of their virulence factors.

Bacterial Species	Virulence Factors	Mechanism	Reference
*orphyromonas gingivalis*	FimA fimbriae	increase proinflammatory cytokines production; mediate the binding to other pathobionts	(Goulbourne and Ellen, 1991; Yamada et al., 2005; Wang et al., 2007)
	Mfa1 fimbriae	increase proinflammatory cytokines production; mediate self-aggregation	(Hajishengallis, 2009; Hajishengallis et al., 2013; Ikai et al., 2015)
	gingipains	damage structural proteins and components; hydrolyze antimicrobial peptides and complement components	(Genco et al., 1999; Popadiak et al., 2007; Bostanci and Belibasakis, 2012; Bryzek et al., 2019)
	LPS	promote the release of MMP-3	(Herath et al., 2013)
	OMVs	carry pathogenic agents; induce proinflammatory factors production	(Uemura et al., 2022)
	capsules	promote biofilm formation; enhance adhesion ability	(Dierickx et al., 2003; Rosen and Sela, 2006)
	chemotactic factors	recruit and activate immune cells	(Dahlstrand Rudin et al., 2020)
	peptidyl arginine deiminase	promote the release of pro-inflammatory cytokines; shield *P. gingivalis* from phagocytosis	(Prucsi et al., 2023)
*Tannerella forsythia*	S-layer protein	promote the adhesion and immune evasion	(Sakakibara et al., 2007; Shimotahira et al., 2013; Philips et al., 2020)
	BspA protein	aggregate with *o*ther pathobionts, develop biofilm	(Sharma, 2010)
	mirolysin	degrade complement components and antimicrobial peptides	(Radisky, 2020)
	karilysin	degrade antimicrobial peptides; induce proinflammatory factor production	(Bryzek et al., 2014; Skottrup et al., 2019)
	serine protease	disrupt periodontal tissues	(Hockensmith et al., 2016)
	OMVs	promote the release of pro-inflammatory cytokines; carry pathogenic agents	(Lim et al., 2023)
	LPS	induce proinflammatory cytokine expression	(Chinthamani et al., 2022)
*Aggregatibacter actinomycetemcomitans*	leukotoxin	dissolve and kill immune cells; impair immune function; promote proinflammatory cytokines release;	(Johansson et al., 2000; Claesson et al., 2002; Johansson, 2011; Balashova et al., 2016; Johansson et al., 2017)
	cytolethal distending toxin	compromise macrophage phagocytic function	(Shenker et al., 2022; Kim et al., 2023)
	outer membrane protein A	promote complement components consumption	(Lindholm et al., 2019; Lindholm et al., 2020)
	OMVs	promote TNF-α production; carry pathogenic toxins	(Han et al., 2019; Nice et al., 2024)
	adhesion proteins	promote biofilm formation	(Danforth et al., 2021)
*Treponema denticola*	sialidase	damage the epithelial barrier; complement system	(Kurniyati et al., 2013; Frey et al., 2019)
	flagellin	promote the motility	(Kurniyati et al., 2017)
	dentipain protease	facilitate adhesion and nutrients acquisition; degrade host proteins	(Ishihara et al., 2010; Miyai-Murai et al., 2023)
	major surface protein	contribute to immune evasion	(Jones et al., 2019)
	dentilisin	upregulate MMP-2 expression; induced IL-6 and HSP70 production; degrade complement proteins	(Miller et al., 2016; Ateia et al., 2018; Kokubu et al., 2018; Malone et al., 2021)
*Prevotella intermedia*	LPS	induce production of pro-inflammatory mediators	(Choi et al., 2021)
	cysteine protease interpain A	breakdown albumin, haemalbumin, and hemoglobin releases heme	(Byrne et al., 2015)
	superoxide reductase	lower the Eh and create anaerobic milieu	(Karched et al., 2022)
*Fusobacterium nucleatum*	LPS	induce production of inflammatory factors	(Vinogradov et al., 2022)
	FadA	facilitate cell adhesion; promote biofilm formation; increase proinflammatory cytokines release	(Li et al., 2020; Meng et al., 2021; Wang Y. et al., 2023)
	Fap2	promote biofilm formation	(Coppenhagen-Glazer et al., 2015)
	RadD	promote biofilm formation	(Engevik et al., 2021)

Various substances associated with oral pathobionts, such as the cell wall component LPS and metabolic products like short-chain fatty acids, exhibit potent biological activity (Huang and Guan, 2023; Park et al., 2023). **Table 1** summarizes the major pathobionts and the mechanisms of action of their virulence factors. As shown in **Table 1**, these metabolites can directly stimulate gingival tissues, triggering local inflammatory responses. For instance, LPS from *P. gingivalis* and *Escherichia coli* can directly stimulate gingival fibroblasts and epithelial cells, initiating an inflammatory response (Yang et al., 2021; Yang et al., 2024). Moreover, these metabolites can suppress the defensive functions of host cells, exacerbating periodontal damage. Butyrate, produced by *P. gingivalis*, inhibits the clearance of inflammatory factors by fibroblasts and promotes their apoptosis, compromising the periodontal barrier (Shirasugi et al., 2017; Tsukahara et al., 2020; Nakagawa et al., 2021). Furthermore, these microorganisms and their products can activate the host’s specific immune response, inducing the production of a plethora of inflammatory mediators, such as cytokines and matrix metalloproteinases. For example, LPS from *P. gingivalis* can activate the NF-κB signaling pathway, leading to the activation of M1 macrophages. M1 macrophages secrete cytokines like IL-1β and TNF-α, which activate osteoclasts and release matrix metalloproteinases, causing the destruction of periodontal tissues (Huang et al., 2019; Xu et al., 2023; Li et al., 2024). The OMVs secreted by *P. gingivalis* can penetrate host tissues, promoting neutrophil infiltration and inducing macrophages to produce pro-inflammatory mediators, thereby exacerbating periodontitis (Fleetwood et al., 2017; De Souza et al., 2022; Kim et al., 2022; Wielento et al., 2022).

### 3.3 Dysbiosis and the host immune response

The local periodontal microenvironment in periodontitis patients exhibits immunological differences compared to healthy individuals. In a healthy state, the host immune system can recognize and eliminate oral pathobionts, maintaining oral health. This involves a balanced interaction between the innate and adaptive immune responses, with antimicrobial peptides and immune surveillance mechanisms effectively controlling microbial balance. When dysbiosis occurs, it triggers the host immune response, leading to inflammation to clear the pathobionts (Shahbeik et al., 2021). However, persistent dysbiosis can overwhelm these regulatory mechanisms, leading to chronic inflammatory response and persistent microbial dysbiosis.

The inflammatory response involves the recruitment and activation of immune cells, which have both pro-inflammatory and anti-inflammatory functions. Immune cells, such as macrophages, neutrophils, and T cells, are not only defensive cells in the progression of periodontitis but can also contribute to the development of inflammation (Marinho et al., 2015; Mishra et al., 2023). On the one hand, the pro-inflammatory factors they secrete, such as IL-1β and IL-6, attract more immune cells to the lesion, prolonging the inflammation (Marinho et al., 2015; Mishra et al., 2023). Additionally, these cytokines can activate osteoclasts, leading to the resorption of alveolar bone and furthering tissue destruction (Alippe et al., 2017). The prolonged inflammatory response can lead to excessive activation and damage to the host immune system, resulting in tissue destruction and pain (Zhang J. et al., 2020). Specifically, the activity of these cells can also affect the balance of the host immune system, promoting detrimental immune regulatory mechanisms. For instance, IL-6 and IL-23 secreted by M1 macrophages can promote the expansion of pathogenic Th17 cells, exacerbating periodontitis (Dutzan et al., 2018). Moreover, the polarization of T cells toward a Th17 phenotype can inhibit regulatory T cells, which are crucial for maintaining immune tolerance and preventing excessive inflammation. Pro-inflammatory cytokines such as IL-1β, IL-6, and IL-8 in the environment of periodontitis stimulate neutrophils to transition into a more aggressive and pro-inflammatory state (Williams et al., 2021). This shift toward a pro-inflammatory phenotype disrupts the balance between effector and regulatory immune responses, leading to the excessive production of inflammatory mediators (Williams et al., 2021). These altered neutrophils are also capable of releasing reactive oxygen species and proteases, which not only exacerbate tissue destruction but also further recruit additional immune cells, amplifying the inflammatory cascade (Williams et al., 2021).

## 4 Therapeutic strategies for periodontitis based on microbial dysbiosis

Recent research on the microbiological mechanisms of periodontitis has demonstrated that its development is not only associated with the presence of specific pathobionts but also closely related to the overall balance of the oral microbiome (Albuquerque-Souza and Sahingur, 2022; Di Stefano et al., 2022; Liu et al., 2023). Based on this concept, novel therapeutic strategies have been developed that aim to prevent and treat periodontitis by regulating and managing the composition of the microbial community.

### 4.1 Targeted therapeutics for microbial dysbiosis

Nonsurgical treatment of periodontitis primarily involves the removal of dental plaque and calculus through scaling and root planning (SRP), often supplemented with local or systemic antibiotic therapy (Khattri et al., 2020). However, the escalating risk of bacterial resistance, coupled with the potential disruption of the beneficial oral microbiota balance due to antibiotic use, presents significant challenges. Additionally, pathobionts can form biofilm structures that protect them from antibiotic attacks, further diminishing the efficacy of treatment. Consequently, strategies for eliminating pathobionts are continually being updated and optimized. Research on therapeutic drugs has focused on developing novel antibiotics, antibiotic delivery systems, and nanoparticles.

SRP stands as the primary treatment for periodontitis, adept at efficiently eliminating plaque from both teeth and periodontal pockets. However, it’s worth noting that certain pathobionts, particularly *A. actinomycetemcomitans*, may continue to persist despite treatment (Takamatsu et al., 1999). Therefore, antimicrobial therapy is often combined with SRP for the treatment of periodontitis in clinical practice. Studies have shown that adjunctive systemic antimicrobial therapy can improve the outcomes of periodontitis treatment (Lu et al., 2022). **Table 2** summarizes the currently used antibiotic therapies for pathobionts. In addition to directly inhibiting pathobionts, these antibiotics can be utilized in various other aspects of periodontitis treatment. For instance, Zhang et al (Zhang et al., 2021). developed a minocycline hydrochloride-loaded microsphere/sucrose acetate isobutyrate hybrid depot, which ensures the sustained release and maintenance of minocycline at appropriate concentrations. At these optimal concentrations, minocycline not only exhibits antimicrobial activity but also promotes osteoblast growth, demonstrating significant potential in the treatment of periodontitis. Similarly, doxycycline, beyond its antibacterial properties, can inhibit the pathological upregulation of MMPs involved in tissue degradation during periodontitis (Mittal et al., 2022). This inhibition helps to mitigate the tissue destruction caused by periodontitis. Furthermore, the combination of antibiotics with natural antimicrobial agents can enhance the efficacy of antibiotics, effectively addressing the rising issue of antibiotic resistance. Ibrahim et al. demonstrated that the combination of metronidazole with extracts from *Symphytum officinale* and *Panax ginseng* significantly enhances the inhibitory effect of metronidazole on *P. gingivalis* biofilms (Ibrahim et al., 2023). Additionally, this combination suppresses the production of acylated homoserine lactones, thereby impairing the bacterium’s quorum-sensing capabilities and ultimately reducing its pathogenicity (Bacali et al., 2022).

**Table 2:** Used targeted antibiotic therapies for pathobionts currently.

Antibiotics	Categories	Mechanism	Anti-microbial	Reference
Minocycline hydrochloride	Tetracycline	Inhibit bacterial protein synthesis and osteogenesis effects.	*P. gingivalis* and *A. actinomycetemcomitans*	(Zhang et al., 2021)
Doxycycline Hyclate	Tetracycline	Inhibit bacterial protein synthesis.	*P. gingivalis* and *A. actinomycetemcomitans*	(Mittal et al., 2022)
Clindamycin	Lincomycin	Inhibit bacterial protein synthesis and enhance neutrophil chemotaxis and phagocytosis	*E. nodatum* and *P. intermedia*	(Luchian et al., 2021)
Metronidazole	Nitroimidazole	Inhibit bacterial DNA replication	*P. gingivalis*	(Ibrahim et al., 2023)
Moxifloxacin HCl	Fluoroquinolone	Inhibit bacterial DNA replication and transcription	*P. gingivalis*	(Senarat et al., 2023b)
Levofloxacin	Fluoroquinolone	Inhibit bacterial DNA replication and transcription	*P. gingivalis, P.macaccae and P. heparinolytica*	(Senarat et al., 2023a)

Antibiotic delivery systems are devices or materials that deliver antibiotics precisely to periodontal pockets, such as polymer nanoparticles and nanogels. These systems are capable of effectively encapsulating antibiotics, offering the advantage of precise drug delivery to targeted sites. Additionally, they facilitate the sustained release of the drug, ensuring that an effective concentration of the medication is maintained over an extended period (Brun et al., 2020). For instance, Lin et al (Lin et al., 2018). developed pH-responsive PLGA/chitosan nanoparticles encapsulating metronidazole, enabling targeted and controlled drug release in acidic environments. This design minimizes the drug’s impact on healthy tissues and prevents initial burst release, enhancing treatment sustainability and safety. The nanoparticles significantly reduce inflammatory responses and alveolar bone loss, and facilitate tissue repair. Additionally, this delivery system effectively preserves beneficial oral microbiota, offering a more efficient and comprehensive solution for periodontitis management.

With the problem of antibiotic resistance caused by antibiotic overuse, new antimicrobial materials are receiving attention. Nanotechnology provides new antimicrobial materials for the oral field, including metal nanoparticles and polymer nanoparticles. Silver nanoparticles (AgNPs), exemplifying metal nanoparticles, can significantly inhibit bacterial growth in oral biofilms associated with periodontitis by disrupting bacterial cell walls and preventing biofilm formation (Hernández-Venegas et al., 2023). In Hernández-Venegas et al’s study, AgNPs were evaluated *in vitro* using biofilms collected from patients with and without periodontal disease, demonstrating that their antimicrobial efficacy increases as particle size decreases (Hernández-Venegas et al., 2023). AgNPs, in particular, exhibit excellent dispersibility and a large surface area, which enhances their contact and binding with bacteria while effectively preventing particle aggregation (Hernández-Venegas et al., 2023). This combination of properties significantly boosts their bactericidal capabilities, making silver nanoparticles a promising antimicrobial material for treating periodontitis. Polymer nanoparticles, such as curcumin nanoparticles, have been shown to exhibit antimicrobial efficacy comparable to 1% chlorhexidine gel in a clinical study involving patients with chronic periodontitis (Guru et al., 2020). In this randomized controlled trial, curcumin nanoparticles effectively improve clinical parameters and reduce the levels of *P. gingivalis*, *T. forsythia*, and *A. actinomycetemcomitans* compared to SRP solely (Guru et al., 2020).

### 4.2 Bacteriophage therapy in periodontitis

Phage therapy is a promising and emerging approach for the treatment of periodontitis. Phage therapy encompasses not only the use of a single bacteriophage but also phage-derived enzymes, phage-antibiotic combinations, synthetic phages, and phage cocktails (Guo et al., 2024). This diversification of phage applications offers more options and possibilities for clinical use.

Compared with conventional antibiotic therapy, phage therapy offers several advantages. Firstly, considering that bacteriophages generally possess a narrow host range, typically exhibiting specificity toward only a limited subset of strains within a single species. Phages are capable of specifically targeting and eliminating pathobionts without causing significant harm to the beneficial oral microbiota, thereby mitigating the risk of microbial dysbiosis. For example, the five novel lytic bacteriophages (JD-Fnp1 ~ JD-Fnp5) isolated by Wang et al (Wang et al., 2022). exhibit specificity in targeting and lysing *F. nucleatum*, resulting in a significant reduction of its abundance within the periodontal microbiome. Furthermore, these bacteriophages do not cause substantial damage to beneficial oral microbial communities, thereby enabling a more effective maintenance and restoration of the healthy balance within the periodontal microecosystem. In past studies, phage therapy has been commonly used to treat biofilm-embedded pathobionts. For example, the biofilm of *Streptococcus mutans* demonstrated a reduction in cell viability by 5 log_10_upon treatment with bacteriophage ϕAPCM01 in an *in vitro* biofilm assay, highlighting its potent anti-biofilm activity (Dalmasso et al., 2015). Tinoco et al (Tinoco et al., 2016). have demonstrated that the engineered bacteriophage ϕEf11/ϕFL1C(Δ36) PnisA can effectively disrupt *Enterococcus faecalis* biofilms, including antibiotic-resistant strains in a vitro study. Additionally, depolymerases encoded by bacteriophages, including enzymes such as sialidases, levanases, and xylosidases, degrade the polysaccharide matrix within the biofilm (Pires et al., 2016). This enzymatic degradation significantly enhances the phage’s ability to penetrate bacterial biofilms. In addition, bacteria are less likely to develop phage resistance compared with antibiotics, further enhancing the clinical application prospects of phage therapy. However, this does not imply that bacteria are incapable of developing resistance to bacteriophages. Bacteria can acquire phage resistance through mechanisms such as receptor gene mutations, phase variation, and epigenetic modifications (Burmeister et al., 2020). To mitigate the potential emergence of phage resistance, the utilization of phage cocktails is primarily recommended to decrease the likelihood of resistance development. Furthermore, the isolation of novel phage strains specifically designed to target resistant bacterial populations serves as an alternative strategy (Khalifa et al., 2015).

### 4.3 Probiotic therapy in periodontitis

Oral probiotics play an essential role in maintaining the stability of oral microbiota. Common oral probiotics include *Lactobacillus*, *Bifidobacterium*, *Streptococcus*, and *Bacillus* (Homayouni Rad et al., 2023). These beneficial microorganisms can suppress the growth of pathobionts through various mechanisms, thereby preserving the dynamic balance of the oral microbial community. Specifically, probiotics can directly inhibit the growth and colonization of pathobionts by secreting antimicrobial substances and competing for nutrients. For instance, *Lactobacillus fermentum* (*L. fermentum*) ALAL020 can inhibit the growth of pathobionts such as *P. gingivalis* by producing a cyclic dipeptide (Kawai et al., 2022). Furthermore, probiotics can create an acidic environment and disrupt harmful bacteria’s cell membranes, enzyme activities, and metabolic processes, thereby further suppressing their growth (Argandoña Valdez et al., 2021). In addition to regulating the balance of the oral microbiome, probiotics can also stimulate the activity of oral immune cells, enhancing the local immune defense against pathogenic microorganisms. *L. acidophilus* La5 downregulates the release of CXCL-8, GM-CSF, and IL-1β by *A. actinomycetemcomitans*-stimulated gingival epithelial cells, modulating local immune responses and inflammation in the gingiva (Bueno et al., 2022). In oral diseases such as periodontitis, the oral beneficial bacteria can become disrupted due to host or environmental factors, leading to the overgrowth of pathobionts and persistent inflammatory responses. Therefore, re-establishing the equilibrium of oral beneficial bacteria is a crucial aspect of periodontitis treatment. Moreover, probiotic-based therapies are gaining attention as an alternative approach in the context of increasing antibiotic resistance.

Unlike conventional antibiotic treatments, probiotic therapy offers the advantage of selectively targeting pathobionts without disrupting the commensal oral microbiota, thereby avoiding microbiome dysbiosis. This selective action and ability to modulate the oral ecosystem make probiotic therapy a valuable adjunct to other innovative treatment modalities, such as phage therapy, in periodontal disease management. Studies have demonstrated that certain probiotic strains possess antimicrobial, anti-inflammatory, and immunomodulatory properties, effectively inhibiting the growth and virulence of pathobionts. For instance, *L. fermentum* ALAL020 exerts growth-inhibitory effects against *P. gingivalis* and *P. intermedia* by producing a cyclic dipeptide (Kawai et al., 2022). *L. acidophilus* La5 and *L. rhamnosus* Lr32 significantly reduce the adhesion/invasion of *A. actinomycetemcomitans* to gingival epithelial cells OBA-9 and decrease the transcription of virulence factors associated with host defense (Ishikawa et al., 2021; Bueno et al., 2022). Furthermore, probiotics can suppress pathobiont growth by counteracting biofilm formation. For example, *L. plantarum* 14917 significantly inhibits the growth of *Streptococcu*s mutans and *Candida albicans* within a multispecies biofilm model (Wu et al., 2015). Concurrently, *L. plantarum* 14917 further disrupts the biofilm structure of the pathogens by lowering the medium’s pH and reducing the biofilm’s dry weight and total protein content (Wu et al., 2015). Probiotics such as *L. acidophilus* JCM 1021 and *L. casei* NBRC 15883 have also been found to inhibit *A. actinomycetemcomitans* biofilm formation (Jaffar et al., 2016). Topical application of probiotic supplements, such as probiotic mouthwashes or toothpaste, has also shown promising clinical efficacy (Parčina Amižić et al., 2017; Seminario-Amez et al., 2017). Additionally, probiotics exhibit anti-inflammatory properties by modulating the host’s immune response. For instance, the experiments conducted by Invernici et al (Invernici et al., 2018). have successfully demonstrated that *Bifidobacterium animalis* subsp. *lactis* HN019 can modulate the inflammatory response in periodontal disease treatment by downregulating pro-inflammatory cytokines IL-1β and IL-8, while upregulating anti-inflammatory factors IL-10 and TGF-β. Furthermore, *Bifidobacterium animalis* subsp. *lactis* HN019 has been reported in another study to enhance the expression of β-defensin 3 and TLR4 in gingival tissues, thereby boosting the activity of CD-4^+^ T cells (Invernici et al., 2020), and then contributing to the reduction of excessive pro-inflammatory cytokine production. This anti-inflammatory effect helps maintain the balance of the oral microenvironment by inhibiting the growth of pathogenic bacteria under inflammatory conditions.

Although probiotic therapy has demonstrated potential in the treatment of periodontitis, current research predominantly focuses on the short-term effects of such treatments, leaving the sustainability of probiotic therapy’s efficacy largely unexplored. The Meta-Analysis conducted by Mendonça et al (Mendonça et al., 2024). revealed that probiotic treatments extending beyond one month did not exhibit sustained benefits, suggesting that providing probiotic therapy for more than one month yields no significant difference. Therefore, future studies should consider extending the follow-up period to assess the long-term effects and potential sustainability of probiotic therapy.

### 4.4 Microbiological considerations in treating periodontitis

Microbiological considerations for periodontitis therapy involve identifying and quantifying pathobionts, assessing pathobiont virulence factors, and predicting post-treatment microbial changes. Dental plaque samples can be collected from periodontal pockets for microbial analysis. Given that a significant portion of the oral microbiome cannot be cultured, techniques such as polymerase chain reaction (PCR) and fluorescence *in situ* hybridization (FISH) can be employed to achieve a more comprehensive understanding of the microbial landscape associated with periodontal disease (Gu J et al., 2022; Murugaiyan et al., 2024). Furthermore, sequence-based culture-independent approaches, such as 16S rRNA gene sequencing and metagenomic sequencing, offer a more detailed analysis of the identification and quantification of unculturable microorganisms (Jünemann S et al., 2012; Shi C et al., 2021). In addition to identifying pathobionts, understanding their virulence factors is crucial. These factors dictate the severity and invasiveness of the disease caused by the pathobionts. Relevant virulence factors include biofilm formation, toxin production, and protease activity, as detailed in Section 2. Because the composition of the periodontal microbiota changes following periodontal therapy, it is important to predict post-treatment outcomes. Typically, there is a decrease in the abundance of pathobionts and an increase in the abundance of beneficial bacteria. However, in some cases, pathobionts may persist or recolonize, leading to treatment failure. Therefore, prediction of post-treatment outcomes is paramount.

These microbiological considerations are essential for treatment plans for periodontitis. Based on pathobionts identification, antibiotics and other antibacterial substances that target specific pathobionts are selected to achieve the goal of precision treatment. Adjunctive use of biofilm disruptors, such as chlorhexidine (Merchant et al., 2022), polysaccharides (Alsalhi, 2024), and hydrogen peroxide (Brookes et al., 2023), can enhance the efficacy of antibacterial substances. Due to the periodontal tissue being reduced during the process of periodontitis, regenerative techniques such as guided tissue regeneration can be used to promote bone and periodontal tissue regeneration (Janjić et al., 2020). Accurate identification and quantification of pathobionts, understanding their virulence factors, and predicting post-treatment microbial changes aid in developing personalized treatment plans, thereby improving treatment outcomes.

## 5 Conclusions and perspectives

### 5.1 Challenges

While we have come to understand that dysbiosis of the oral microbiome is a key factor in the pathogenesis of periodontitis, there are still challenges in its practical application. Firstly, the oral microbiome is highly complex and dynamic (Peng et al., 2022). The oral cavity harbors a multitude of bacteria that interact in intricate ways, making it difficult to comprehensively monitor and modulate the microbiome. Additionally, the microbiome is subject to dynamic changes influenced by factors such as age, diet, and lifestyle, adding to the difficulty of precise modulation (Li et al., 2021). Concurrently, the composition of the microbiome varies among individuals (Nearing et al., 2020). These differences are not only reflected in the types and abundances of microorganisms but also may influence responses to antimicrobial treatments. This leads to variability in therapeutic outcomes, yet concurrently highlights the advantages of precise treatment. Current periodontitis treatment modalities, such as mechanical debridement and antimicrobial agents, often overlook the role of the oral microbiome and fail to fully restore microbial homeostasis (Kwon et al., 2021). Thus, there is a need for the development of more targeted and effective therapeutic strategies. The emergence of antimicrobial resistance due to the prolonged use of antimicrobial agents poses a significant challenge to treatment. Therefore, the overuse of antimicrobial agents should be curtailed (Yuan et al., 2023).

For probiotic therapy, the critical hurdles include identifying the optimal probiotic strains that can effectively colonize the oral cavity and compete with pathogenic bacteria (Seminario-Amez et al., 2017; Bustamante et al., 2020). It is also essential to ensure the viability and targeted delivery of these probiotics to the periodontal tissues, as well as maintaining the long-term colonization of the beneficial microbes (Zidar et al., 2021). In the case of phage therapy, the challenges lie in isolating phages with the appropriate specificity to target the pathogenic bacterial species involved in periodontitis and successfully delivering and maintaining therapeutic concentrations of the phages at the site of infection (Shlezinger et al., 2017; Szafrański et al., 2017; Chen et al., 2021). Furthermore, addressing the unique regulatory and safety considerations for the clinical implementation of phage therapy is a critical step before widespread adoption. A pivotal aspect inherent in strategies targeting oral microbial therapeutics lies in the intricate relationship between the oral microbiome and the microbiomes of other bodily sites, such as the gut. Treatments aimed at modulating the oral microbiome may inadvertently affect the microbial communities in the gut, potentially leading to unintended health consequences. Consequently, understanding these interconnections is crucial for developing holistic and safe therapeutic approaches.

### 5.2 Perspectives

Future research and therapeutic strategies should center on personalized and precision medicine approaches. The standard clinical treatment for periodontitis involves the combination of SRP with systemic antibiotics. However, the highly complex nature of the oral microbiome makes comprehensive monitoring and regulation of microbial communities particularly challenging. Consequently, there is a pressing need to develop personalized microbiome profiling and precision treatment plans. Given that the majority of oral microorganisms are difficult to culture, strain identification typically relies on advanced techniques such as PCR and metagenomics. Nevertheless, the high costs associated with these methods remain a significant barrier to their widespread adoption. Future research should aim to reduce the costs of microbiome analysis while simultaneously enhancing its accuracy and efficiency. Additionally, in-depth investigations into the inter-individual variations in microbiomes and their influencing factors will provide a scientific foundation for devising more precise therapeutic strategies.

Moreover, emerging therapeutic strategies, including probiotic therapy and phage therapy, hold promise as alternative treatments. However, translating these approaches into clinical practice presents numerous challenges. Regarding probiotic therapy, existing studies are predominantly short-term, and the long-term efficacy and safety of probiotics remain unclear (Mendonça et al., 2024). Therefore, future efforts should focus on optimizing the screening and delivery technologies for probiotics to ensure their effective colonization and long-term maintenance within the oral environment, thereby enabling the assessment of their sustained therapeutic benefits. Phage therapy has demonstrated effectiveness against various microbial infections, but its application in the treatment of oral diseases has been scarcely explored (Łasica et al., 2024). Additionally, there is a limited availability of phages that specifically target oral pathobionts. Consequently, future research should prioritize the discovery of phages with specificity toward periodontitis-associated pathobionts and address challenges related to phage screening, delivery, and clinical safety. Furthermore, establishing comprehensive regulatory frameworks and conducting large-scale clinical trials are essential steps to facilitate the clinical application of phage therapy.

### 5.3 Conclusion

This review presents a robust and detailed analysis of the role of microbial dysbiosis in periodontitis and explores innovative therapeutic strategies to address this condition. However, the imitations of this review mainly include challenges in translating innovative therapies from the lab to clinical practice, insufficient depth in exploring host-microbiome interactions and immune responses, and a lack of consideration for geographical and demographic variations that could affect microbial dysbiosis and treatment outcomes. Despite these limitations, the elucidation of microbial dysbiosis as a central mechanism in periodontitis paves the way for transformative therapeutic strategies that transcend conventional treatments. By harnessing the power of personalized medicine and microbiome engineering, it is possible to develop precise interventions that not only treat but also prevent periodontitis. Continued research and interdisciplinary collaboration are essential to translate these innovative approaches from the laboratory to clinical practice, ultimately improving oral health outcomes and enhancing the quality of life for patients.
